# New application of bimetallic Ag/Pt nanoplates in a colorimetric biosensor for specific detection of *E. coli* in water

**DOI:** 10.3762/bjnano.15.9

**Published:** 2024-01-17

**Authors:** Azam Bagheri Pebdeni, Mohammad N AL-Baiati, Morteza Hosseini

**Affiliations:** 1 Nanobiosensors lab, Department of Life Science Engineering, Faculty of New Sciences & Technologies, University of Tehran, Tehran, Iran; 2 Department of Chemistry, College of Education for Pur Science, University of Kerbala, Karabal, Iraq; 3 Department of Pharmaceutical Biomaterials and Medicinal Biomaterials Research Center,Faculty of Pharmacy, Tehran University of Medical Sciences, Tehran, Iran

**Keywords:** aptamer, biosensor, detection, *E. coli*, peroxidase activity, silver/platinum nanoplate

## Abstract

A fast and sensitive aptasensor was developed using nanoplates with peroxidase activity as a novel approach*. E. coli* detection is described using a silver/platinum nanoplate (Ag/Pt NPL) that interacts with an oligonucleotide aptamer as a bioreceptor. The size of the Ag/Pt NPLs was about 42 nm according to the FE-SEM images. The EDS result indicates that a thin layer of Pt ions was coated on the surface of the Ag NPLs. This nanobiosensor has the ability to specifically bind to *E. coli*, increasing the peroxidase activity of the apt-Ag/Pt NPL. Finally, the blue color of the solution in the contaminated water samples was increased in the presence of 3,3′,5,5′-tetramethylbenzidine (TMB) as a substrate and H_2_O_2_. The assay can be completed in 30 min and the presence of *E. coli* levels can be distinguished with the naked eye. The absorbance at 652 nm is proportional to pathogen concentration from 10 to 10^8^ CFU·mL^−1^, with a detection limit of 10 CFU·mL^−1^. The percent recovery for the water samples spiked with *E. coli* is 95%. The developed assay should serve as a general platform for detecting other pathogenic bacteria which affect water and food quality. The proposed *E. coli* detection strategy has appealing characteristics such as high sensitivity, simple operation, short testing time, and low cost.

## Introduction

One of the most dangerous foodborne infections is caused by *E. coli.* It can induce hemorrhagic colitis, hemolytic uremic syndrome, and thrombocytopenic purpura*. E. coli* is responsible for more than 2 million acute illness cases worldwide each year [1–2]. Water quality testing is based on the presence of *E. coli*, which is still regarded as the best indication of fecal contamination [3–4]. There are several methods for detecting bacteria (e.g., ELISA and PCR) with colony counting serving as the gold standard. However, these procedures need a significant amount of time, skilled operators, and costly equipment. As a result, for effective medical treatment with minimal detection time, a selective and sensitive detection strategy is required [5–6]. New nanoparticles for fast bacterial detection can be fabricated.

**Scheme 1:**
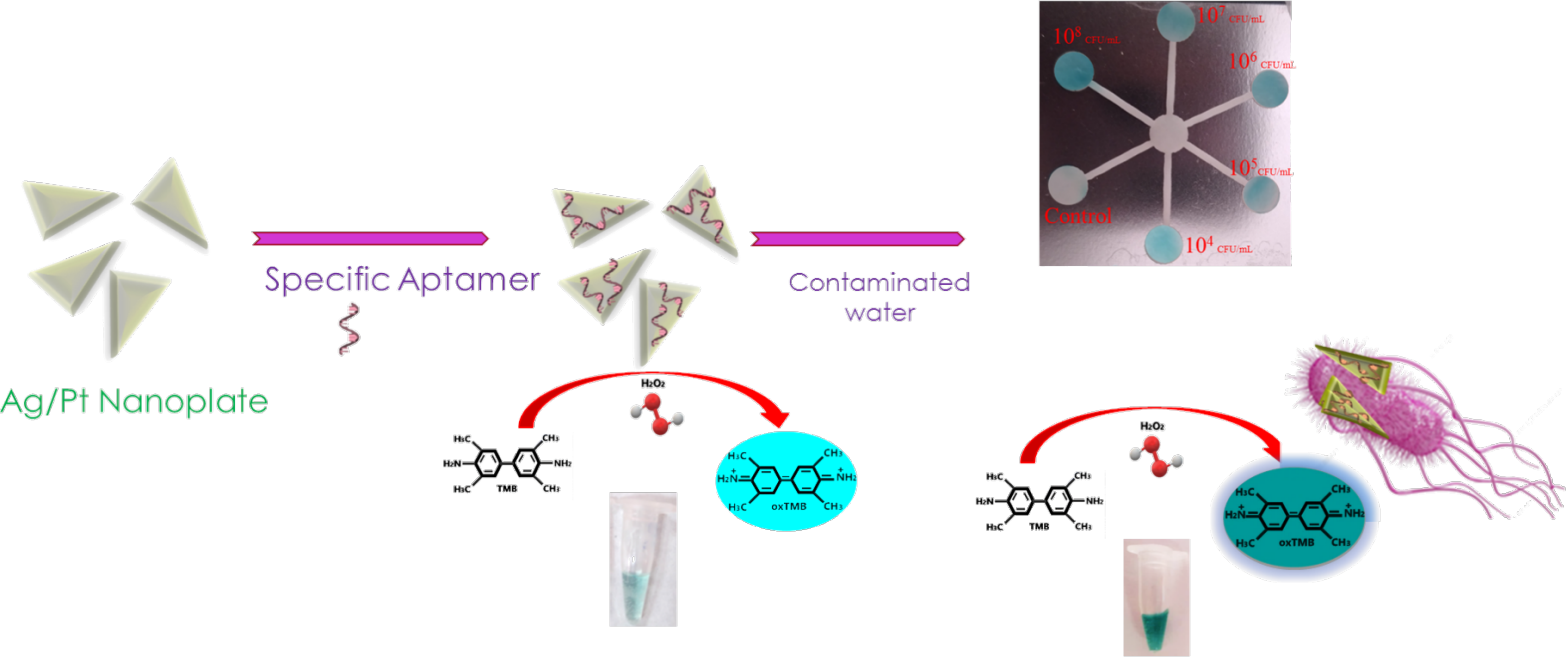
Schematic representation of the detection of *E. coli* by Ag/Pt NPL as a peroxidase nanozyme in solution and paper based microfluidic device.

Colorimetric biosensors have become popular in analytical applications due to their high sensitivity, convenience, and ease of signal readout [7–8]. These biosensors have been extensively utilized in pathogen identification, primarily because of their ability to rapidly display results in visible color [9–10]. Nanomaterials have improved the ability to detect pathogens in water and food by enhancing signals and sensitivity. These materials, which encompass nanoparticles, nanorods, nanowires, and nanoclusters, can be combined with aptamers, antibodies, enzymes, and other ligands to display unique physical, chemical, and optical traits. The colorimetric method typically depends on the enzymatic properties, aggregation, and dispersion of these nanomaterials, which can be influenced by the presence of aptamers, antibodies, and other agents [4]. Among the various colorimetric test techniques, the enzymatic conversion of a chromogenic substrate, such as 3,3′,5,5′-tetramethylbenzidine (TMB), is widely employed. This conversion process can generate vibrant products when H_2_O_2_ is present [11–12]. Colorimetric biosensors often use chromogenic substrates such as TMB, ABTS, and *o*-phenylenediamine (OPD) to produce a visual readout signal through peroxidase mimetic activity. The TMB is preferred due to its reliance on a single organic substrate, eliminating the need for a helper molecule. Studies have shown TMB to be non-mutagenic, but it may still be carcinogenic. However, the low solubility of TMB in water requires modifications to increase its solubility [7]. Due to its low cost, simplicity, fast response, and the lack of expensive equipment required, the peroxidase-like activity has garnered significant attention in the detection of harmful microorganisms. The naked eye can easily observe the blue hue resulting from the catalytic oxidation of TMB by H_2_O_2_ on a paper-based device. As a result, it is highly suitable for the generation of novel and portable biosensors [13]. The color shift produced by an enzyme-catalyzed substrate reaction is an appealing option for developing colorimetric-based biosensors to detect targets such as pathogens [14]. Functional nanomaterials with catalytic activity similar to enzymes (nanozymes) reveal substantial benefits over natural enzymes, such as ultrahigh environmental stability, appropriate catalytic activity, and ease of prototyping [15–16]. We created plate-like silver nanoparticles (i.e., silver nanoplates, Ag NPLs) covered with a layer of Pt atoms to improve the peroxide activity of NPLs, and use them as colorimetric biosensor materials. Metallic NPLs were employed in a variety of applications, including antibacterial activity [17–19], hazardous dye removal [20], photocatalytic degradation and bactericidal action [21], sensors and biosensors [22–25], and as electrocatalysts [26]. Aptamers are single-stranded DNA or RNA oligonucleotides that attach to their targets with great affinity and specificity. Aptamers have high stability in a variety of environments and during long-term storage and can be manufactured using normal oligonucleotide chemical synthesis processes, with minor chemical changes if necessary. Aptamers are thus regarded as a good baroreceptor in a variety of biological applications [27–28]. Colorimetric paper-based biosensors are the most popular and appealing since the presence of a specific pathogen can be easily observed by a simple change in color, which can be distinguished easily with the naked eye eliminating any use of expensive and complex types of equipment [29–31]. In this research, we provide a novel and highly sensitive nanobiosensor as well as a paper-based analytical equipment for detecting *E. coli*. The *E. coli* could be trapped by the aptamer-NPL to create bacteria–aptamers–Ag/Pt NPL complexes in which the aptamers effectively change on the surface of Ag/Pt NPL. Surprisingly, in the conventional TMB and H_2_O_2_ color reaction, the bacteria–aptamers–Ag/Pt NPL complex displayed a substantially greater color yield (Scheme 1). The aptamer-NPL complex was able to distinguish *E. coli* from other foodborne pathogens, as evidenced by a significantly different signal. This detection principle was applied to contaminated water, where *E. coli* was successfully detected using the developed colorimetric sensor. The sensor offers a straightforward, sensitive, and dependable method for detecting pathogens and ensuring water safety.

## Results and Discussion

### Characterization of Ag/Pt NPL and apt-NPL

**Figure 1:**
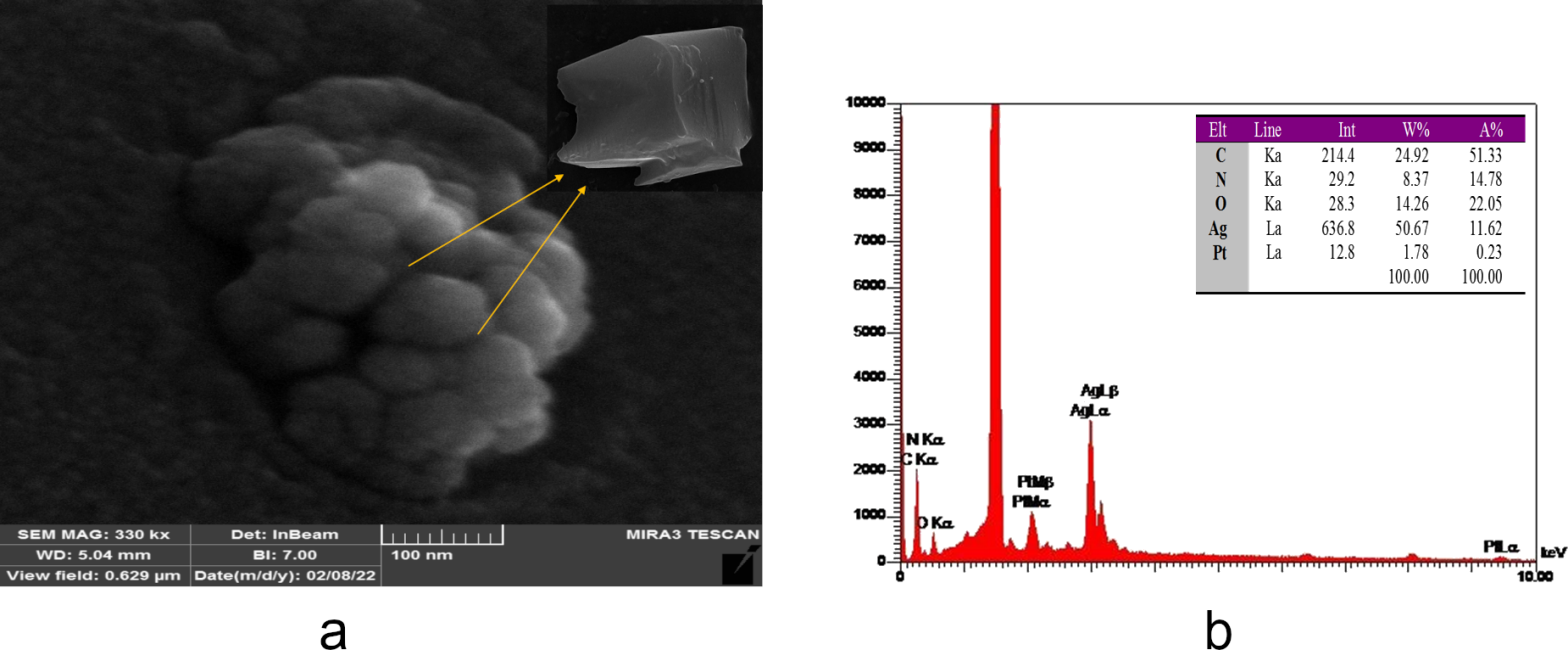
a) The SEM image AND b) EDS analysis of Ag/Pt NPL.

Field-emission scanning electron microscopy (FE-SEM) was used to investigate the form and shapes of NPLs. The size of the NPLs is approx. 42 nm. Figure 1a shows a combination of truncated triangular and circular plates of Ag/Pt NPLs. The NPLs were evenly distributed and shaped in the form of discs or triangles. Energy-dispersive X-ray spectroscopy (EDS) was used to examine Ag and Pt on the synthesized NPLs (Figure 1b). The percentage of Pt is smaller than that of Ag, indicating that a thin layer of Pt ions has been coated on the surface of Ag NPLs. In the aqueous solution, the Ag/Pt NPLs appear bluish purple. To predict the secondary structure of the oligonucleotides the UNAfold program was employed, as illustrated in Supporting Information File 1, Figure S1.

### Peroxidase-like activity of Ag/Pt NPLs

**Figure 2:**
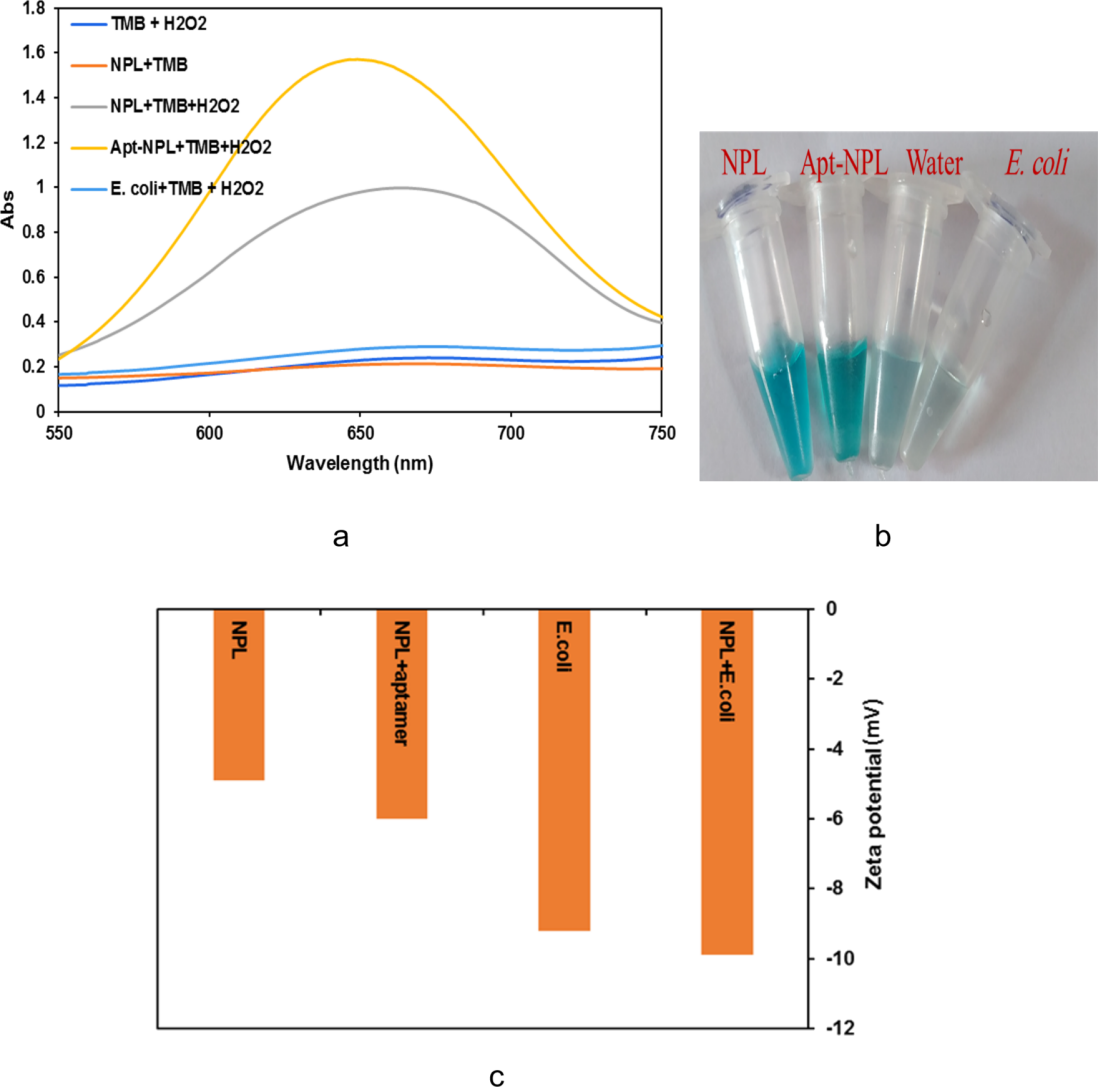
a) Absorbance spectra of Ag/Pt NPL after the addition of TMB, TMB and H_2_O_2_, NPL, and aptamer-NPL in the presence of TMB-H_2_O_2_. b) Image related to the color change of the proposed NPL, c) the zeta potential of NPL, aptamer-NPL, *E. coli* bacteria, and NPL-*E. coli*.

Supporting Information File 1, Figure S2a illustrated a comparison of the inherent peroxidase activity of Ag/Pt NPLs. The intrinsic peroxidase activity of Ag/Pt NPLs is substantially greater than those of silver or platinum alone. When compared to Ag NPLs or Pt NPLs alone, the bimetallic NPLs demonstrated higher peroxidase-like activity. We synthesized Ag NPLs and Pt NPLs under the same conditions as bimetallic NPLs. After adding TMB and H_2_O_2_, we compared the peroxidase activity of the three NPLs at 652 nm. The catalytic activity of Ag NPLs, Pt NPLs, and Ag/Pt NPLs demonstrate the superiority of bimetallic NPLs over the others in terms of peroxidase-like catalytic activity. These nanostructures were successfully used to make a colorimetric aptasensor for *E. coli* sensing. The substrate used to examine the peroxidase-like activity of the Ag/Pt NPLs was TMB since it may be oxidized by H_2_O_2_ to create a blue-colored product during the peroxidase-like catalysis. The Ag/Pt NPLs were synthesized by reducing Ag and Pt using ascorbic acid as the reducing agent in the synthesis of NPs. The chromogenic TMB was oxidized in the presence of Ag/Pt NPLs, resulting in a noticeable blue hue. As shown in Supporting Information File 1, Figure S2a, the absorbance intensity at 652 nm, which is the characteristic peak of oxidized TMB, considerably rose after the addition of Ag/Pt NPLs. The best concentration of TMB and H_2_O_2_ that resulted in the maximum peroxide activity of NPLs throughout the optimal period of 4 min was chosen (Supporting Information File 1, Figure S2b). Different concentrations of TMB were used as the substrate when the H_2_O_2_ concentration was adjusted to 1 M. As indicated in Supporting Information File 1, Figure S3a, 10 mM of TMB has the maximum peroxide activity of NPL throughout 4 min. The varied concentrations of H_2_O_2_ (0.5–3 mM) were examined after the concentration of TMB was set at 10 mM. The peroxide activity of NPL is maximum at 1 M H_2_O_2_ (Supporting Information File 1, Figure S3b). The stability of the NPLs in different buffers was tested and the results showed suitable peroxidase activity of this nanozyme in various solutions (Supporting Information File 1, Figure S3c). The shelf-life of the aptasensor is at least six months; storage at 4 °C is necessary. The peroxide activity of the Ag/Pt NPLs enhanced following the addition of the aptamer. Using DNA to functionalize NPLs can increase not only target recognition but also enzyme activity, which is a useful technique for developing biosensors for target detection. Furthermore, the electrostatic and intermolecular interactions of aptamer–TMB increased the substrate affinity of NPLs. As a result, the catalytic efficacy of Ag/Pt NPL was improved, resulting in a deeper blue signal (Figure 2a, Figure 2b) [32–33]. The zeta potentials of Ag/Pt NPLs, aptamer-NPLs, aptamer-NPLs coupled with *E. coli*, and *E. coli* alone were measured (Figure 2c). The surface charges of all NPLs were negative due to dispersion agents such as sodium citrate and ascorbic acid, according to measurements of their zeta potentials. The NPLs had a negative charge of −4.4 mV before introducing the aptamers, which changed to −6 mV. A reduction in the zeta potential of the aptamer-Ag/Pt NPLs revealed that the aptamer was effectively changed on the surface of NPLs. In comparison to *E. coli* and aptamer-NPLs, the negative charge of aptamer-NPL-*E. coli* reduced to −10 mV after incubation time, indicating that the particular aptasensor was gathered around the whole cell of *E. coli*, implying that aptamer-NPLs might successfully capture the target bacterium.

### Principles of *E. coli* detection

**Figure 3:**
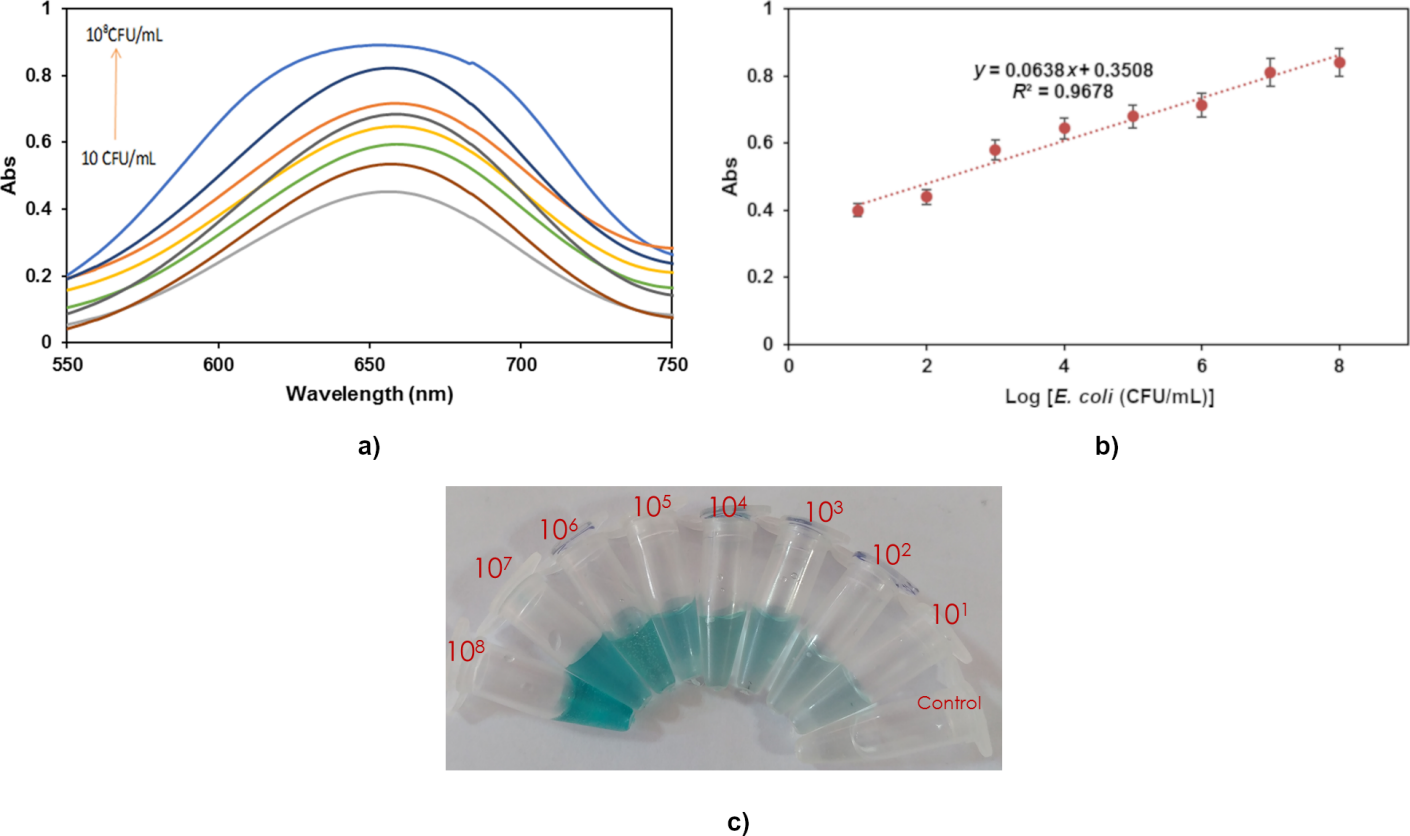
a) The calibration curve of different concentrations of *E. coli* (10 to 10^8^ CFU·mL^−1^) obtained at 652 nm. b) Linear relationship of the aptamer-Ag/Pt NPL for detection of *E. coli*, and c) images showing color change.

After adding TMB and H_2_O_2_, a particular quantity of *E. coli* was introduced to the produced aptamer-Ag/Pt NPLs, and the changes in peroxidase-like activity were measured. According to Figure 3a and Figure 3b, the peroxidase-like activity of NPLs was enhanced after the addition of a particular target to the aptamer-NPL solution. At the same time, the control was light blue. This technique indicated that the aptamer, as a specific bioreceptor immobilized on the NPLs, specifically attached to *E. coli*. The standard curve was generated in concentrations ranging from 10 to 10^8^ CFU·mL^−1^ using the linear regression equation *y* = 0.0638*x* + 0.3508 (*R*^2^ = 0.9678). The sensitivity of the NPLs was studied, and its detection limit (LOD) was calculated to be 10 CFU·mL^−1^ (*S*/*N*) after a 30 min detection time with the naked eye, indicating that the concentration of *E. coli* in the sample was growing (Figure 3c).

If the sample color is too subtle, we can enter the amount of gray intensity of the sample in the calibration curve and calculate the concentration of the target in the tested sample. The color of the sample must always be checked against the control. The aptamer-Ag/Pt NPL solution can greatly increase the intensity of the distinctive peak of the catalytic system. The bacteria were introduced to a solution containing TMB and H_2_O_2_ to validate the phenomenon, and no alterations in color were detected, as shown in Figure 2 (light blue color curve). These findings showed that *E. coli* lacked peroxidase activity. However, the introduction of bacteria in the catalytic aptasensor can enhance the creation of the blue product. The resultant agent is generated by the specific binding of the aptamer to the surface of the bacteria and the accumulation of numerous biosensors around the target leads to an increase in the intensity of the blue color. As a result, we attempted to explain the phenomenon and used it to develop a biosensor for detecting pathogens. To qualify the concentration of bacteria, the blue product in the solution was employed as a signal reporter. This study also demonstrated that enriching bacteria with aptamer-NPL can increase peroxidase-like activity. Table 1 compares the proposed colorimetric aptasensor to different previously published *E. coli* detection methods. The findings were compared to those of previous specified situations, proving that this technique gives an increased dynamic range and appropriate LOD. This biosensor offers several advantages such as quick reaction, high sensitivity, ease of use, low cost, and the capacity to do analysis for an extended period of time. Also, this aptasensor can function well on a paper-based platform and, as the cost of the paper is low, the production of this biosensor compared to many similar biosensors is lower.

**Table 1:** Comparison of the analytical performances between the method presented in this paper and the reported methods for the detection of *E. coli*.

Method	Nanostructure	Bioreceptor	Analytical range (CFU·mL^−1^)	LOD (CFU·mL^−1^)	Ref.

SERS-LFA strips	core–shell of Au–Ag	antibody	6.94 × 10^1^–10^9^	6.94 × 10^1^	[34]
fluorescent	Yb,Tm,Fe-doped NaYF4 NPs	label-free	100–10^9^	36	[35]
colorimetric	papain-AuNCs	aptamer	10^2^-10^6^	5.6 × 10^2^	[36]
immunochromatographic assay	Au/Pt NP	mAb	1.93 × 10^4^–1.93 × 10^8^	1.83 × 10^4^	[37]
colorimetric	magnetic probe	T7 bacteriophage	–	1 × 10^4^	[38]
fluorescent	GO-Fe_3_O_4_	aptamer	10^3^–10^7^	467	[39]
colorimetric paper-based microfluidic device	Ag/Pt NPL	aptamer	10–10^8^	10	this work

### Evaluation of selectivity

The selectivity of the Ag/Pt NPLs for *E. coli* was examined under the same conditions with *Staphylococcus aureus*, *Salmonella typhimurium*, *Bacillus subtilis*, and *Pseudomonas aeruginosa*. The findings demonstrated that non-specific strains could not be adhered to the aptamer immobilized on the NPLs. As a result, their absorbance was greater than that when *E. coli* was present (Supporting Information File 1, Figure S4). The NPL selectivity refers to the specificity of the applied aptamer toward the surface structures of the target. The aptamer boosted the NPL selectivity and specificity against *E. coli*, and it is not prone to adhering to other bacteria.

### Paper-based colorimetric detection of *E. coli* in water as a real sample

**Figure 4:**
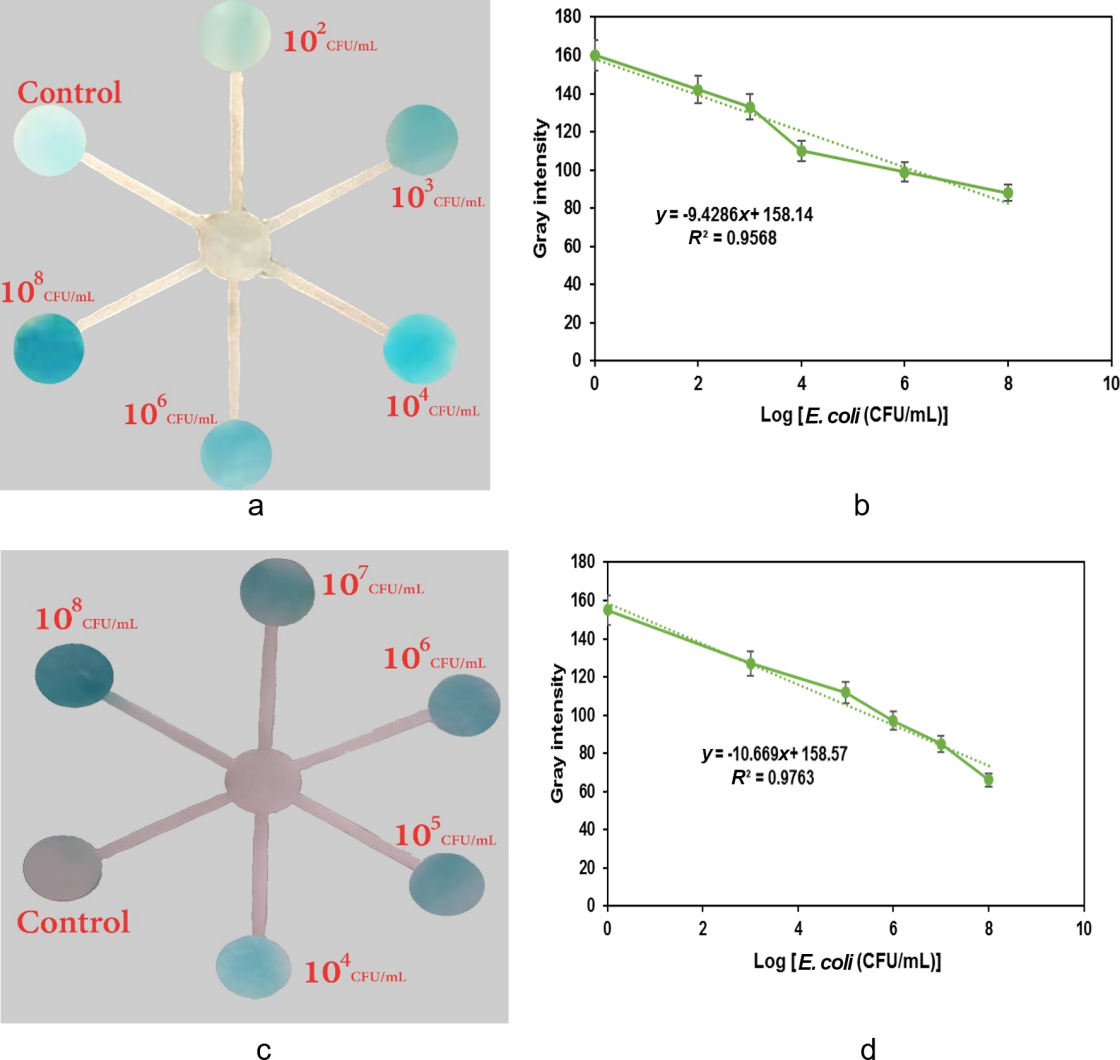
(a, c) Analytical results of the paper-based model for naked eye detection of *E. coli*, (b, d) the gray intensity of grey value intensity increased at different concentrations of the target in a, b) tap water and c, d) lake water.

The suggested biosensor paper-based model was meant to monitor the change in blue color with different concentrations of *E. coli*. The vivid blue hue showed an increase in bacteria concentrations after the addition of TMB and H_2_O_2_ to the paper-chip containing the aptasensor and *E. coli*. Due to the increase in aptamer–bacteria interaction based on paper-chip pores, this paper-based biosensor was not time-dependent and showed a fast color-change response in the absence or presence of specific target (Figure 4). The intensity of the blue color, calculated with the ImageJ software, shows a desirable linear correlation with the logarithm of *E. coli* concentration, as shown in Figure 4. In the equations, ''*y*'' was the intensity of the response value and ''*x*'' is the log concentration of *E. coli*. The color was blue, and the *E. coli* concentration was high. The outcomes confirmed this. Each paper-based sensor is usable once, but the preparation of the paper-based sensor is not complex and shows suitable reproducibility. This paper-based method can accurately distinguish between different *E. coli* concentrations, making it a quick and convenient method for testing.

Tap water and lake water were chosen as candidates to evaluate the potential of this colorimetric biosensor in real sample analysis. The lake water sample was collected from Bandar Anzali wetlands (Gilan, Iran). The *E. coli* presence was examined by spiking known amounts of the bacteria in water samples. *E. coli* thresholds in drinking water is well below 10^4^ CFU·mL^−1^, which is the limit of the colorimetric test [36]. Thus, our biosensor is very sensitive for detection of *E. coli* in drinking water and for analyzing the food safety. The percent recovery of the target bacteria was calculated as the ratio of the concentration of the spiked target bacteria in the real samples to the bacteria concentration in the method (measured concentration). The relative standard deviation (RSD) was obtained by multiplying the standard deviation by 100 and dividing the result by the average. As shown in Supporting Information File 1, Table S1, the recovery percentage ranged from 94 to 103 percent, emphasizing the effectiveness and accuracy of the biosensor in detecting *E. coli* in drinking water. A paper platform was optimized to execute the aptamer-based assay with the goal of producing a fast and portable biosensor. The paper-based microfluidic system can result in an increased sensitivity due to the shorter amount of sample used without any need for dilution, separation, or pre-treatment through various stages.

## Conclusion

In this research, Ag/Pt NPLs with outstanding peroxidase-like activity for the detection of *E. coli* in contaminated water sample were synthesized. The combination of catalytic activity and aptamer interaction allows for the specific detection of *E. coli*, as shown by an increase in the blue color intensity which correlates with bacterial concentration. By utilizing paper-based analysis, we were able to achieve naked-eye detection without the need for instruments. Additionally, the peroxidase-like activity of the NPLs paper-based colorimetric sensor was suggested for the first time. With a low detection limit of 10 CFU·mL^−1^, this system offers a rapid, sensitive, and portable biosensor for preventing *E. coli* contamination and resolving public health concerns in the future.

## Experimental

### Materials

Silver nitrate (AgNO_3_), potassium tetrachloroplatinate(II), ascorbic acid, TMB, H_2_O_2_ (for determining peroxidase-like activity), and sodium hydroxide were purchased from Sigma. Tris-EDTA and HCl (for TE buffer production) were purchased from Merck. The aptamer for identifying *E. coli* (5′-CCC CCC CCC CCG GAC GCT TAT GCC TTG CCA TCT ACA GAG CAG GTG TGA CGG-3′) was acquired from Pishgam (Tehran, Iran). Lyophilized bacterial strains and bacterial culture medium were supplied from Baharafshan Institute of Research and Development (Tehran, Iran). All other substances were of analytical reagent grade and were used as supplied, with no further purification. The treatment was carried out with sterile deionized water.

### Apparatus

Perkin-Elmer lambda 25 UV–vis spectrometer was employed for UV–vis absorption measurement in the range of 200–800 nm. The morphology and shape of NPLs were studied using a field-emission scanning electron microscope (Supra 400VP, Zeiss, Oberkochen, Germany). Dynamic light scattering experiments were carried out at room temperature using a Zetasizer Nano-ZS90 Malvern. The elementary compositions of the samples were determined using EDS on a Tescan energy-dispersive spectrometer. The ImageJ software was used to determine the gray intensity of the blue color of the paper-based microfluidic device.

### Synthesis of Ag/Pt NPLs

A modified chemical reduction method was used to create Ag NPLs [26,38]. The aqueous solution synthesis of Ag-Pt NPL is described below. To begin, 50 L of 0.05 M AgNO_3_ aqueous solution was mixed with 10 mL of 2.5 × 10^−4^ M sodium citrate aqueous solution. After that, 25 µL of 0.1 M ascorbic acid solution was gradually added to a stirred mixture of sodium citrate and AgNO_3_. The purple Ag seed solution was then obtained. In addition, 10 mL of 0.05 M AgNO_3_ aqueous solution was mixed with 20 mL of 0.1 M hexadecyltrimethylammonium bromide (CTAB) aqueous solution. Slowly, 10 mL of 0.1 M ascorbic acid and 0.408 mL of the prepared Ag seed solution were dropped into the CTAB aqueous solution. The Ag nanotemplates were circular. After adding 0.8 mL of 2 M NaOH aqueous solution, the circular Ag nanotemplates were prepared. To lessen the interaction of free CTAB with the synthesis of the triangular Ag-Pt NPLs, 200 mL of the synthesized Ag circular NPLs was precipitated by centrifugation at 4000 rpm and redispersed in DI water (3 mL). To complement the Ag NPLs with Pt, at a fixed controlled temperature (60 °C), 8.3 µL of K_2_PtCl_4_ solution was added to 3 mL of stirred Ag NPL solution. The circular Ag-Pt NPLs were obtained after 70 min.

### Bacterial culture

In a similar manner to what has been show in reference [5], Gram-positive and negative strains of pathogenic bacteria such as, *S. aureus* (ATCC 29213), *E. coli* (ATCC 35218), *Pseudomonas aeruginosa* (ATCC 10145), *Salmonella typhimurium* (ATCC 14028), and *Bacillus subtilis* (ATCC 168) were cultured in sterile Luria-Bertani broth (LB broth) and incubated at 37 °C overnight. After centrifugation, the bacteria were transferred to nutrient agar plates and cultured for 24 h at 37 °C. The absorbance of several bacterial colonies of diverse strains was measured using UV–vis spectroscopy at a wavelength of 600 nm (OD600). The gold standard method of plate counting was used to estimate the number of bacterial cells.

### Detection of *E. coli* for bacterial assays

Following the optimization of the experimental settings, various concentrations of *E. coli* (10–10^8^ CFU·mL^−1^) were combined with the aptamer-Ag/Pt NPL solution and gently shaken at room temperature. The peroxidase activity of NPLs in the presence of TMB and H_2_O_2_ was evaluated using a UV–vis spectrophotometer after the incubation period was shortened.

### Analysis in real samples

Tap water and lake water were used as real samples to evaluate the analytical efficacy of the aptasensor. To do this, different quantities of *E. coli* were spiked in real samples which were previously sterilized. Finally, the spiked samples were mixed with the aptasensor and incubated for 30 min as the optimum incubation time. The peroxidase-like activity of the aptasensor was ultimately demonstrated in the presence of TMB and H_2_O_2_.

### Paper-based device

Whatman filter paper No. 1 is considered as an appropriate paper for paper-based analysis. A PVC sheet was affixed to the back of each paper to prevent solution penetration through the bottom of the sheets (similarly to reference [12]). To conduct the analysis, 3 µL of aptamer-NPL was dropped on the paper in the first stage, and after drying, this procedure was repeated two more times to get a total amount of 9 µL of aptamer-NPL on each deposition zone. Following that, one drop of the *E. coli*-contaminated sample was put onto a paper chip. The final stage in the paper evaluation was to add 6 µL of H_2_O_2_ and 6 µL of TMB to paper chips. The ImageJ software was utilized to evaluate the intensity of the resulting blue color.

## Supporting Information

Supporting Information contains the simulated structures of the aptamers and additional experimental information.

File 1 Additional figures and tables.
