# Supplementary material for: New application of bimetallic Ag/Pt nanoplates in a colorimetric biosensor for specific detection of E. coli in water
Source: Beilstein J Nanotechnol. 2024 Jan 17;15:95–103. doi: 10.3762/bjnano.15.9 (PMC10804531; doi:10.3762/bjnano.15.9)
Supplement: File 1 — Additional figures and tables. [file Beilstein_J_Nanotechnol-15-95-s001.pdf]

BEILSTEIN JOURNAL OF NANOTECHNOLOGY

Supporting Information

for

New application of bimetallic Ag/Pt nanoplates in a colorimetric biosensor for specific detection of *E. coli* in water

Azam Bagheri Pebdeni, Mohammad N. AL-Baiati and Morteza Hosseini

Beilstein J. Nanotechnol. **2024,** *15,* 95–103. doi:10.3762/bjnano.15.9

Additional figures and tables

License and Terms: This is a supporting information file under the terms of the Creative Commons Attribution License (https://creativecommons.org/licenses/by/4.0). Please note that the reuse, redistribution and reproduction in particular requires that the author(s) and source are credited and that individual graphics may be subject to special legal provisions.
The license is subject to the *Beilstein Journal of Nanotechnology* terms and conditions: (https://www.beilstein-journals.org/bjnano/terms)

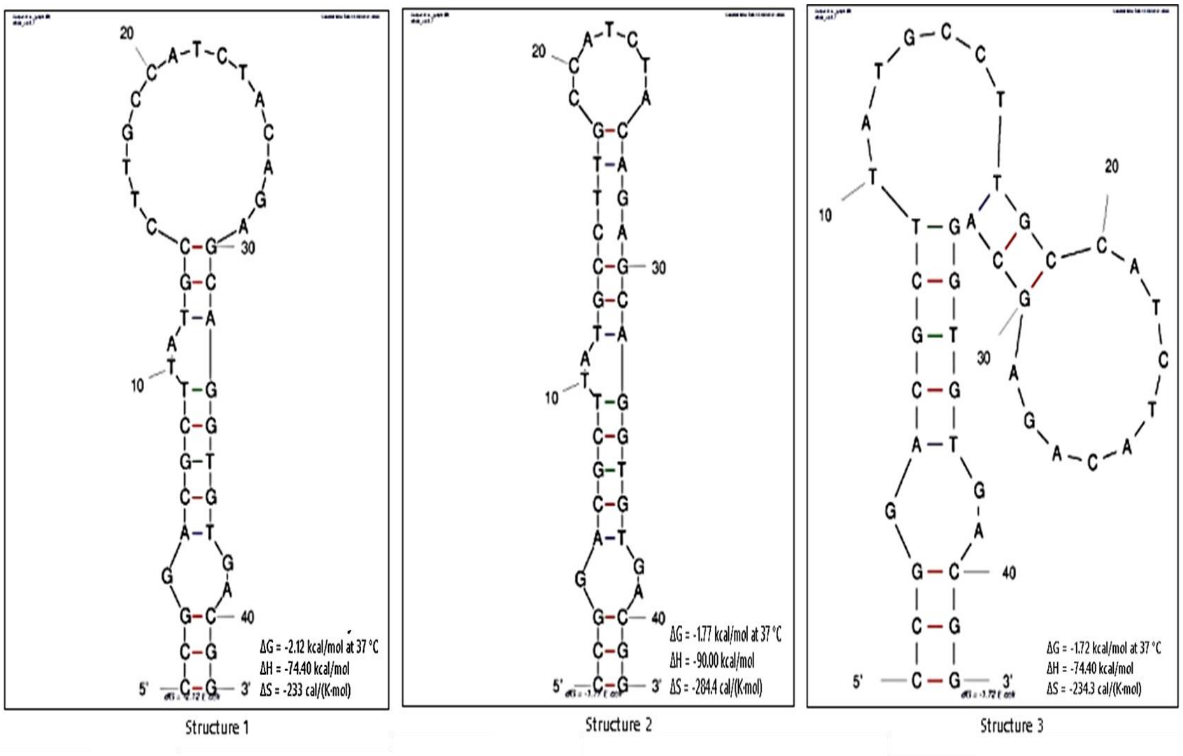

Figure S1: The predicted secondary structure of the sequence of specific aptamers of *E. coli*.

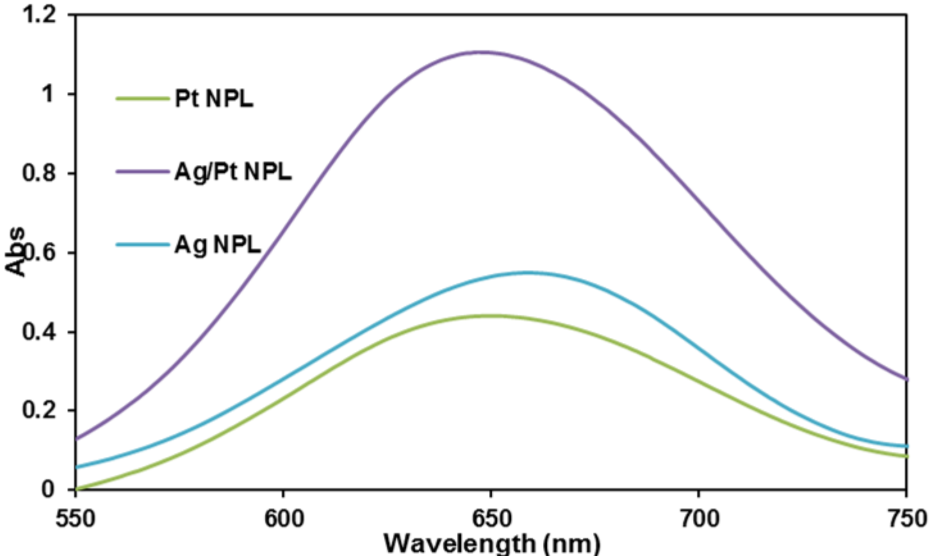

a

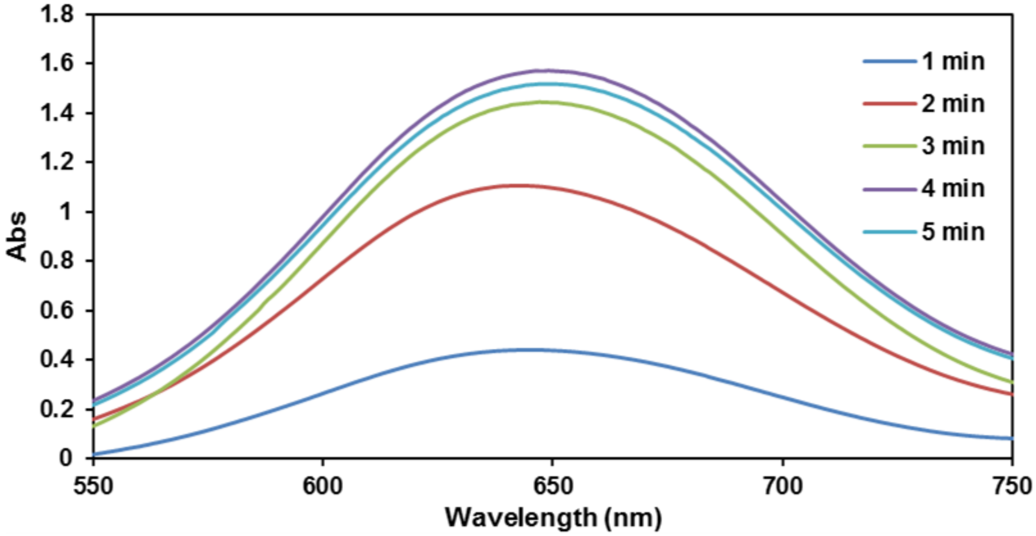

b

Figure S2: a) Comparison of peroxide activity of Ag NPL, Pt NPL, and Ag/Pt NPL after addition of TMB and H_2O_2. b) The peroxidase activity of Ag/Pt NPL after addition of TMB and H_2O_2.

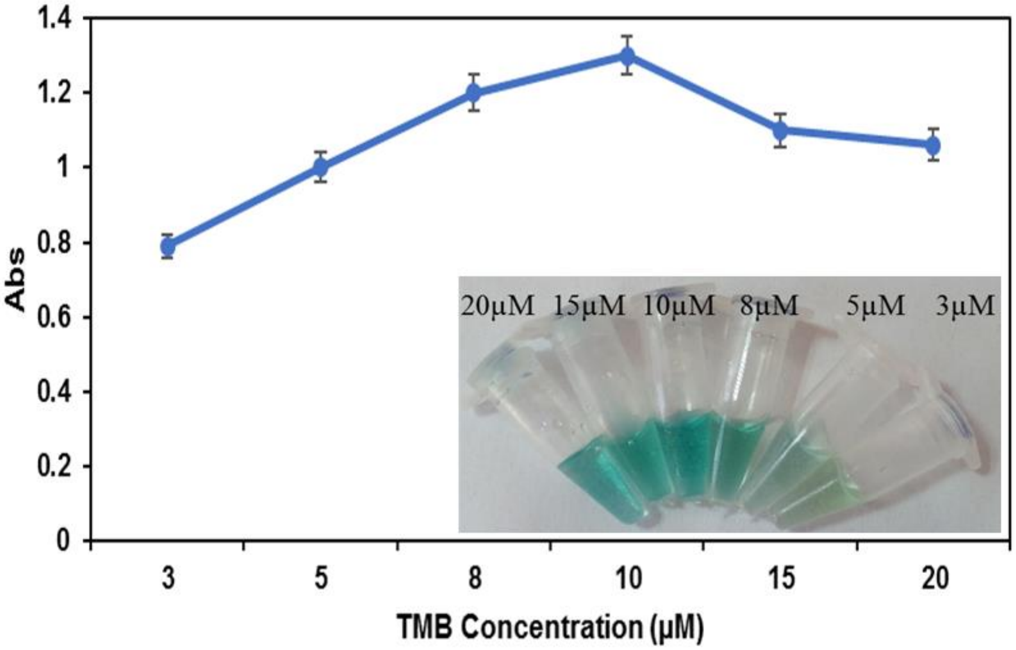
1.4
1.2
1
0.8
0.6
0.4
0.2
0
Abs
3
5
8
10
15
20
TMB Concentration (µM)
20µM
15µM
10µM
8µM
5µM
3µM

a

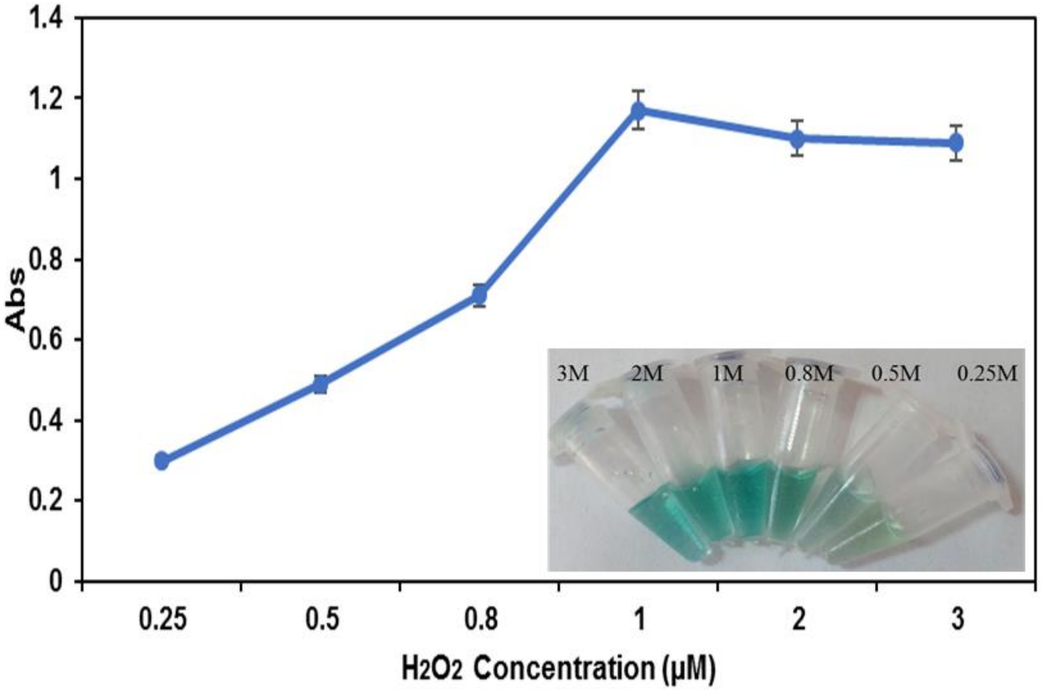
1.4
1.2
1
0.8
0.6
0.4
0.2
0
Abs
0.25
0.5
0.8
1
2
3
H2O2 Concentration (µM)
3M
2M
1M
0.8M
0.5M
0.25M

b

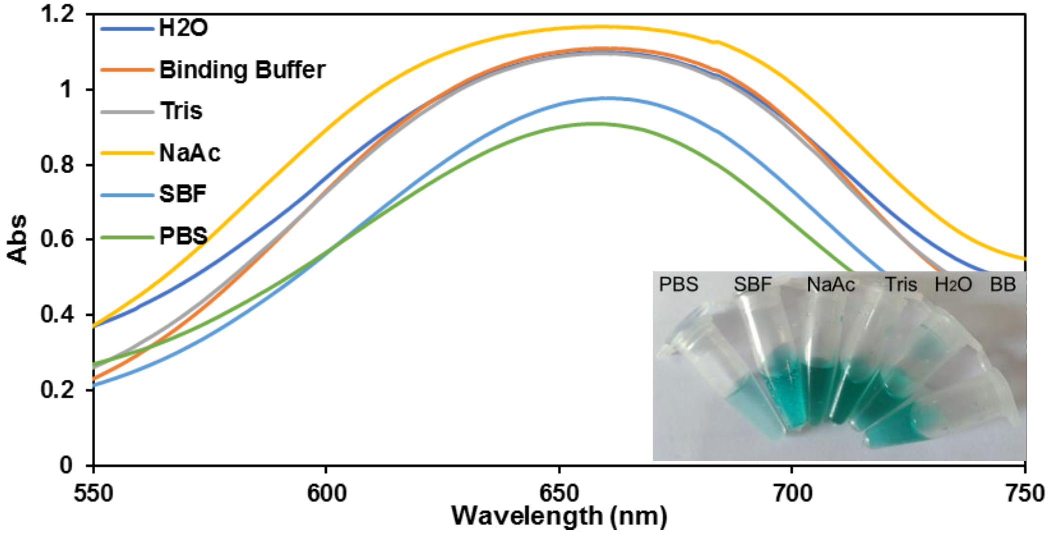

c

Figure S3: Catalytic optimization analysis of Ag/Pt NPL in different concentrations of a) TMB (3, 5, 8, 10, 12 mM) (H_2O_2 = 1 M), b) H_2O_2 concentration (0.5, 1, 2, 3 M) (TMB = 10 µM). c) Stability of peroxide-like activity of NPL in different buffers.

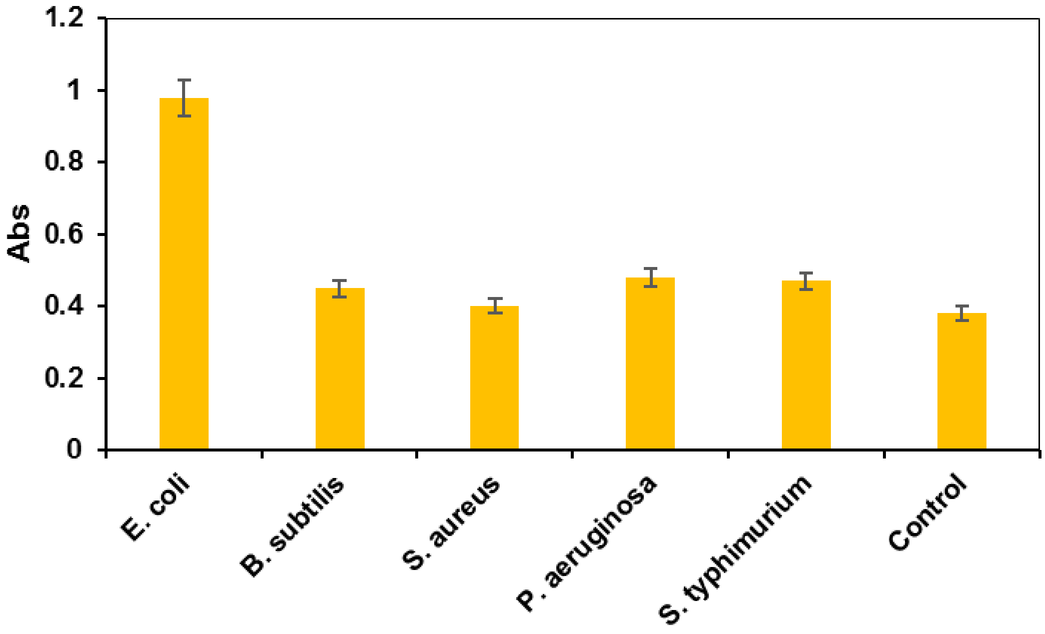

Figure S4: The selectivity of the aptamer-NPL by the same concentration of different bacteria (10^8 CFU·mL^{-1}).

Table S1: Analytical results of different concentration of *E. coli* in drinking water.

Sample	Spiked concentration (CFU·mL^{-1})	Measured concentration (CFU·mL^{-1})	Recovery (%)	RSD (n = 3, %)
Tap water	1×10^{7}	0.95×10^{7}	95	2.3
Tap water	1×10^{5}	0.94×10^{5}	94	3.7
Tap water	1×10^{3}	103×10^{3}	103	1.9
